# Altering FAK-Paxillin Interactions Reduces Adhesion, Migration and Invasion Processes

**DOI:** 10.1371/journal.pone.0092059

**Published:** 2014-03-18

**Authors:** Thérèse B. Deramaudt, Denis Dujardin, Fanny Noulet, Sophie Martin, Romain Vauchelles, Ken Takeda, Philippe Rondé

**Affiliations:** 1 CNRS, UMR 7213, Laboratoire de Biophotonique et Pharmacologie, Illkirch, France; 2 Université de Strasbourg, Faculté de Pharmacie, Illkirch, France; King's College London, United Kingdom

## Abstract

Focal adhesion kinase (FAK) plays an important role in signal transduction pathways initiated at sites of integrin-mediated cell adhesion to the extracellular matrix. Thus, FAK is involved in many aspects of the metastatic process including adhesion, migration and invasion. Recently, several small molecule inhibitors which target FAK catalytic activity have been developed by pharmaceutical companies. The current study was aimed at addressing whether inhibiting FAK targeting to focal adhesions (FA) represents an efficient alternative strategy to inhibit FAK downstream pathways. Using a mutagenesis approach to alter the targeting domain of FAK, we constructed a FAK mutant that fails to bind paxillin. Inhibiting FAK-paxillin interactions led to a complete loss of FAK localization at FAs together with reduced phosphorylation of FAK and FAK targets such as paxillin and p130Cas. This in turn resulted in altered FA dynamics and inhibition of cell adhesion, migration and invasion. Moreover, the migration properties of cells expressing the FAK mutant were reduced as compared to FAK^-/-^ cells. This was correlated with a decrease in both phospho-Src and phospho-p130Cas levels at FAs. We conclude that targeting FAK-paxillin interactions is an efficient strategy to reduce FAK signalling and thus may represent a target for the development of new FAK inhibitors.

## Introduction

In many cancers, progression of the disease results predominantly from the formation of metastases. FAK is involved in many aspects of the metastatic process including adhesion, migration, secretion of MMPs (matrix metalloproteinases) and invasion. Indeed, numerous reports have described overexpression, hyperphosphorylation and/or elevated activity of FAK in a variety of human cancers, including sarcomas, astrocytomas and carcinomas of the breast, colon, thyroid, prostate, oral cavity, liver, stomach and ovary [1]. These observations highlight a possible key role of FAK in tumourigenesis. The first experimental proof implicating FAK in tumour formation and progression was obtained by using conditional knock-out mice with selective *fak* deletion in the epidermis [2]. This proof of concept experiment served as the cornerstone for the development of strategies aimed at inhibiting FAK activity using small-interfering RNAs [3] or small molecule inhibitors. For the latter class, almost all compounds, including PF-562,271 [4], PF-573,228 [5] or TAE226 [6], developed by pharmaceutical companies are ATP-competitive tyrosine kinase inhibitors of FAK. Nevertheless, as FAK possesses both catalytic and scaffolding functions, an alternative possibility to inhibit FAK signalling is to block the adaptor function of FAK. This has been successfully achieved using a small molecule that targets the binding site of FAK and VEGFR3, resulting in suppressed breast cancer growth *in vivo* in mouse models [7].

FAK is a ubiquitously expressed nonreceptor cytoplasmic tyrosine kinase composed of an N-terminal FERM (band 4.1, ezrin, radixin, moesin homology) domain, a central kinase domain, several proline-rich domains and a C-terminal focal adhesion targeting (FAT) domain. The C-terminal domain interacts with focal adhesion (FA)-associated proteins including paxillin and talin [8], [9], p130Cas [10], Grb2 [9], ASAP1 [11] and p85α of PI3K [12]. Furthermore, the C-terminal domain is both necessary and sufficient for localization of FAK to FAs. Structural studies have revealed that FAK targeting to FAs is mediated via FAK-paxillin interactions and to a lesser extent, via FAK-talin interactions. The FAT (Focal Adhesion Targeting) domain of FAK is a four helix bundle containing a large hydrophobic core stabilized by paxillin binding [13], [14]. The 2 paxillin-binding sites present in the FAT domain consist of surface exposed hydrophobic patches (HP). HP1 is located at the surface of helix 2–3 whereas HP2 is located at the surface of helix 1–4. Early experiments using replacement of the FAT sequence of FAK demonstrated that recruitment of FAK to FAs is essential for its regulation by integrin signalling [15]. Moreover, experiments using FRNK (Focal adhesion kinase-Related Non Kinase), the dominant negative form of FAK, which displaces FAK from adhesion sites indicate that many aspects of FAK function require FAK targeting to FAs. Indeed, when overexpressed in cells, FRNK acts as a negative regulator of FAK activity, inhibiting phosphorylation of FAK and various FAK-related processes, including cell cycle progression [16], [17], cell spreading on fibronectin and migration [18], [19]. Overexpression of FRNK in v-Src-transformed NIH3T3 fibroblasts inhibited cell invasion and blocked experimental metastases in nude mice [20]. These data are consistent with displacement of FAK from FAs having a crucial role in FAK signalling-mediated invasion-related processes such as adhesion, migration, invadopodia formation and MMP secretion.

The aim of the present study was to assess the effects resulting from inhibition of FAK-paxillin interactions. Using a mutated form of FAK that does not bind paxillin, we show for the first time that this mutant causes reduction of adhesion, migration and invasion, to a greater extent than that observed in FAK^-/-^ cells demonstrating gain of function effects. Our findings demonstrate that targeting specific FAK-paxillin interactions may be of potential therapeutic interest. Development of such site-specific inhibitors might represent a promising strategy to prevent tumour metastasis.

## Materials and Methods

### Reagents and antibodies

Dulbecco's modified Eagle's medium (DMEM), AlexaFluor 555-conjugated goat anti-mouse IgG and Lipofectamine 2000 were from Invitrogen. Fetal bovine serum (FBS), penicillin, streptomycin and trypsin-EDTA solutions were from Lonza. ReBlot Plus stripping reagent and monoclonal anti-cortactin anti-body (Ab) (clone 4F11) were from Millipore. Human fibronectin, mouse monoclonal anti-FAK kinase, mouse monoclonal anti-phospho-paxillin (Y118) and mouse monoclonal anti-p130Cas Abs were from BD Biosciences. Rabbit anti-phospho-Y925 FAK Ab was from US Biological. Mouse monoclonal anti-paxillin, rabbit polyclonal anti-phospho-Y397 FAK, rabbit anti-phospho-Y576 FAK and rabbit anti-phospho-Y861 FAK Abs were from Invitrogen. Mouse monoclonal anti-β-actin and talin Abs were from Sigma. Mouse monoclonal anti-Src, rabbit polyclonal anti-phospho-p130Cas (Y410) and anti-Y416-phospho-Src Abs were from Cell Signaling. Horseradish peroxidase-conjugated goat anti-mouse or anti-rabbit IgG were from Promega. Rhodamine Red X-conjugated goat anti-mouse, rhodamine Red X-conjugated mouse anti-rabbit, and CY5-conjugated donkey anti-mouse Abs were from Jackson ImmunoResearch Labs.

### Expression vectors

pAcGFP1-Hyg-C1-FAK (human wild-type FAK fused to Cter of GFP) was constructed by inserting the FAK PCR amplified insert in the BglII/SalI restriction sites of pAcGFP1-Hyg-C1 (Clontech). The I936E-I998E-FAK (FAK^I936/I998^) double mutant was generated using the QuikChange II XL site-directed mutagenesis kit (Agilent Technologies). Briefly, pAcGFP1-Hyg-C1-FAK was used as a template to generate the FAK^I936/I998^construct using FAKI936E-S (5′-GCCTGGTGAAAGCTGTCGAGGAGATGTCCAGTAAAATCCAGC-3′), FAKI936E-AS (5′-GCTGGATTTTACTGGACATCTCCTCGACAGCTTTCACCAGGC-3′), FAKI998E-S (5′-GAACTCTGACCTGGGTGAGCTCGAAAACAAGATGAAACTGGCC-3′), and FAKI998E-AS (5′-GGCCAGTTTCATCTTGTTTTCGAGCTCACCCAGGTCAGAGTTC-3′) primers. pcDNA3.1-mCherry-SrcY530F was generated by inserting the PCR product mCherry-SrcY530F in pcDNA3.1(zeo) (Invitrogen). All constructs were amplified and purified using Qiagen Hispeed Maxiprep kits and specific point mutations were verified by sequencing.

### Cell line, transfection and fibronectin stimulation

Primary FAK^-/-^ mouse embryonic fibroblasts (MEFs) [21] were maintained in DMEM supplemented with 10% FBS, 100 U/ml penicillin and 100 μg/ml streptomycin as previously described [22]. Transfection of primary FAK^-/-^ cells with wild type or mutant FAK fused to GFP were performed using Lipofectamine 2000 (Invitrogen) according to the manufacturer's directions. To generate stable populations, 48 h after transfection 150 μg/ml hygromycin was added to allow selection of the transfected cells. After 1 week of hygromycin selection, cells were sorted by FACS. For cells expressing mCherry-Src^Y530F^, FAK^-/-^ and WT or mutant GFP-FAK cells were transfected using Lipofectamine 2000, selected by zeocin for 10 days, and sorted by FACS. For fibronectin stimulation, cells were serum-starved for 24 h, and then seeded onto 10 μg/ml fibronectin precoated culture dishes.

### Cell lysis, immunoprecipitation, and immunoblotting

30 min after stimulation on fibronectin, cells were rinsed 2x with ice-cold PBS (pH 7.4) and lysed with ice-cold IP lysis buffer (137 mM NaCl, 1% Nonidet P-40, 20 mM Tris-HCl, pH 8.0, glycerol 10%, 3 mM Na_3_VO_4_, protease inhibitor tablet [Complete mini, Roche]). Lysates were cleared by centrifugation and protein concentrations determined. For immunoprecipitation, cells were first co-transfected with either FAK-GFP or FAK^I936/I998^-GFP and CFP-paxillin or GFP-talin using Lipofectamine 2000. 500 μg of cell lysates were then incubated with specific Abs (at dilutions recommended by manufacturers) for 3 h at 4°C with continuous shaking. Protein G sepharose beads (Amersham) were then added for overnight incubation. Beads were collected, washed 3x with ice-cold IP buffer and then resuspended in Laemmli buffer. For Western blots, cells were rinsed 2x with ice-cold PBS (pH 7.4) and lysed for 30 min on ice with RIPA buffer. 20 μg of protein lysates were resolved by SDS-10% PAGE and transferred to PVDF membranes (Hybond-P, GE Healthcare). Blocking of membranes was done in 5% nonfat milk-TBST (10 mM Tris-HCl, pH 7.4, 150 mM NaCl, 0.1% Tween 20) for 1 h at room temperature before overnight incubation at 4°C with primary Abs (diluted 1/1000 in 5% nonfat milk-TBST). After 3 washes with TBST, membranes were incubated with corresponding horseradish peroxidase-conjugated secondary Abs (1/20000). Signals were assessed using enhanced chemiluminescence (ECL Plus, GE Healthcare) and CL-XPosure films (Fisher Scientific).

### Adhesion, migration and invasion assays

For adhesion assays, 2.5×10^4^ cells were seeded in fibronectin-coated 96-well plates, allowed to adhere for 1 h before being washed in PBS/0.1% BSA. Cells were then fixed with 4% paraformaldehyde for 10 min, washed and stained with crystal violet (5 mg/ml in 2% ethanol) for 10 min at room temperature. After 5 washes with H_2_O, the dye was extracted from the cells with 0.2% Triton X-100 and optical densities (ODs) measured at 595 nm. Percentages of adhesion were normalized to control cells. For migration assays, cells were seeded on fibronectin-coated IBIDI μ-dishes until confluent. Cell layers were then scratched with pipette tips and cells were allowed to migrate for 8 h. Images of cells were taken at t = 0 and t = 8 h and analysed as previously reported [23].

For migration and invasion assays using mCherry-SrcY530F expressing cells, cells were seeded in migration or matrigel-coated invasion chambers (BD Biosciences) and processed according to the manufacturer's protocol. Migrating or invading cells were fixed with 4% PFA, stained with crystal violet and ODs measured at 595 nm.

### Immunofluorescence microscopy

MEFs were plated at low density on 10 μg/ml fibronectin-coated imaging μ-dishes. After 24 h incubation, cells were fixed with 4% PFA for 10 min, permeabilized in 0.1% Triton X-100 for 5 min, blocked in 1% BSA/PBS for 1 h and incubated with primary Abs diluted at 1/100 to 1/300 in 1% BSA/PBS for 1 h at room temperature. After 3 washes with PBS, cells were incubated with rhodamine-conjugated donkey anti-mouse Ab (1/400), Alexa 555-conjugated goat anti-mouse Ab (1/250) or CY 5 conjugated donkey anti-rabbit Ab (1/250) for 1 h, washed 3 times with PBS and then observed using either a Bio-Rad 1024 confocal system coupled to Nikon Eclipse TE300 microscope (60× CFI Pl Fluor 1.3 NA objective) or a Leica confocal microscope TSC SPE (63x HCX Pl Apo 1.40 NA objective) or a Leica DMIRE2 microscope (40x HCX Pl Apo 1.25 NA and 63x HCX Pl Apo 1.32 NA objectives). For dual wide-field-TIRF experiments cells were imaged using an iMIC microscope (Till Photonics) equipped with a Cobolt Dual Calypso Laser 491/532 nm, a monochromator Polychrome V and an Olympus 60x TIRFM (1.45 NA) objective.

Automated counting of FAs in single cells was done after noise removal by thresholding and applying a size constraint to FAs using ImageJ software. To analyse the percentage of cells expressing ventral adhesions, TIRF images were used and cells with less than 5% of ventral adhesions were counted as negative. For cortactin staining, cells were trypsinized 48 h after transfection and replated on 10 μg/ml fibronectin-coated glass coverslips for 3 h. Cells were then fixed with 4% PFA in PBS for 20 min, permeabilized in 0.5% Triton X-100 for 30 min, blocked in 0.05% BSA in PBS for 30 min and incubated with monoclonal anti-cortactin Ab diluted (1/250) in PBS-BSA for 1 h at 37°C. After 3 washes, cells were incubated with CY5-conjugated donkey anti-mouse Ab (1/350) for 1 h at 37°C, washed 3 times with PBS and mounted in Prolong Gold mounting media (Invitrogen).

### TIRF experiments

For live cell imaging, confluent cells plated on fibronectin-coated coverslips were scratched with a pipette tip prior to imaging. TIRF (total internal reflection fluorescence) images were then acquired using an iMIC microscope (Till Photonics) equipped with a Topica iBeam laser (442 nm) and an Olympus 60x TIRFM (1.45 NA) objective. During acquisition, cells were maintained at 37°C in a 5% CO_2_ humidified atmosphere using an environmental control system (Life Imaging Services). TIRF images were acquired every 1 min on an EMCCD camera (Andor Technology) for 1 h and analysed using ImageJ software.

### Statistical analysis

Data were analysed using either Student's t-test or one way ANOVA followed by post-hoc Newman-Keuls for multiple comparisons. Two ways ANOVA followed by Bonferroni post-tests were used to analyse the size distribution of FAs in the three cell lines. Differences were considered to be significant at p≤0.05. Unless otherwise stated, all data presented are from at least 3 independent experiments.

## Results

### Expression of mutated FAK in the FAT domain alters FAK localization and interaction with paxillin

**Figure 1:**
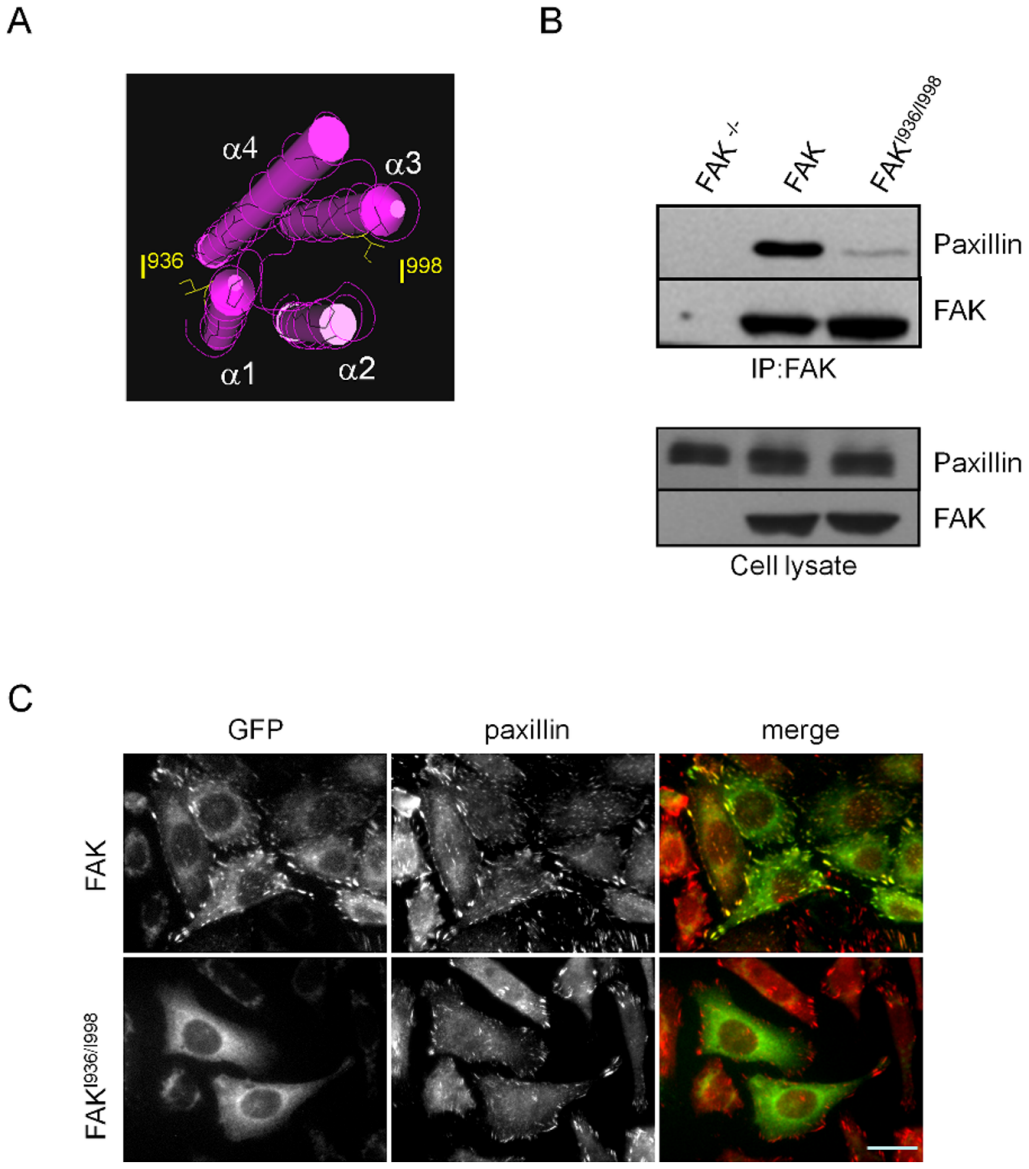
Analysis of FAK-paxillin interaction in wild-type FAK and FAK^I936/I998^ cells. (A) Structural view of the 4 helix bundle (α1-α4) of the FAT domain. Paxillin binds to 2 hydrophobic patches located at the interface of α-helix 1/4 and the interface of α-helix 2/3. The I936E and I998E mutations are indicated. (B) Representative blots showing wild-type FAK and FAK^I936/I998^ immunoprecipitated using anti-FAK Ab and blotted for paxillin and FAK. The expression level of proteins in the corresponding cell lysate is shown. (C) Confocal images from fixed cells expressing wild-type or mutant FAK (fused to GFP) and immunostained for a marker of FAs, paxillin (red). Note the absence of FAK at FAs in mutant cells. Scale bar, 20 μm.

In order to investigate how disruption of FAK-paxillin interactions affects FAK phosphorylation and downstream signalling pathways, site-directed mutagenesis was utilized to replace Ile^936^ and Ile^998^ with glutamic acids Glu^936^ and Glu^998^ in the FAT domain of FAK. These point mutations, for which both HP1 and HP2 are mutated (Figure 1A), have been shown in vitro to completely abrogate binding of truncated paxillin to the FAT domain of FAK [13]. After confirmation of mutations by sequencing, FAK^-/-^ MEFs were transfected with wild-type FAK or FAK^I936/I998^ tagged with GFP. Both proteins were correctly expressed at their expected molecular weights. Co-immunoprecipitation experiments were done to verify that these mutations disrupt FAK interaction with paxillin. As shown in Figure 1B, mutant FAK^I936/I998^ was almost completely unable to precipitate paxillin contrarily to wild-type FAK, thus confirming the requirement of intact HP sites in the FAT domain for FAK-paxillin binding. In order to visualize how these mutations alter FAK localization at FAs, immunofluorescence labelling of MEFs expressing wild-type FAK or FAK^I936/I998^ was done. While FAs were clearly identified in both cell lines by paxillin staining, the FAK mutant is localized into the cytoplasm and not at FAs as shown by the absence of merged signals of FAK with paxillin (Figure 1C). On the other hand, as talin interacts with both FAK and FAK^I936/I998^ and appears correctly localized at FAs in both cell lines (Figure S1), talin may not be an alternative route to localize FAK at FAs thus confirming recent studies [24].

### Expression of FAK^I936/I998^ alters the phosphorylation states of FAK and FAK downstream substrates

**Figure 2:**
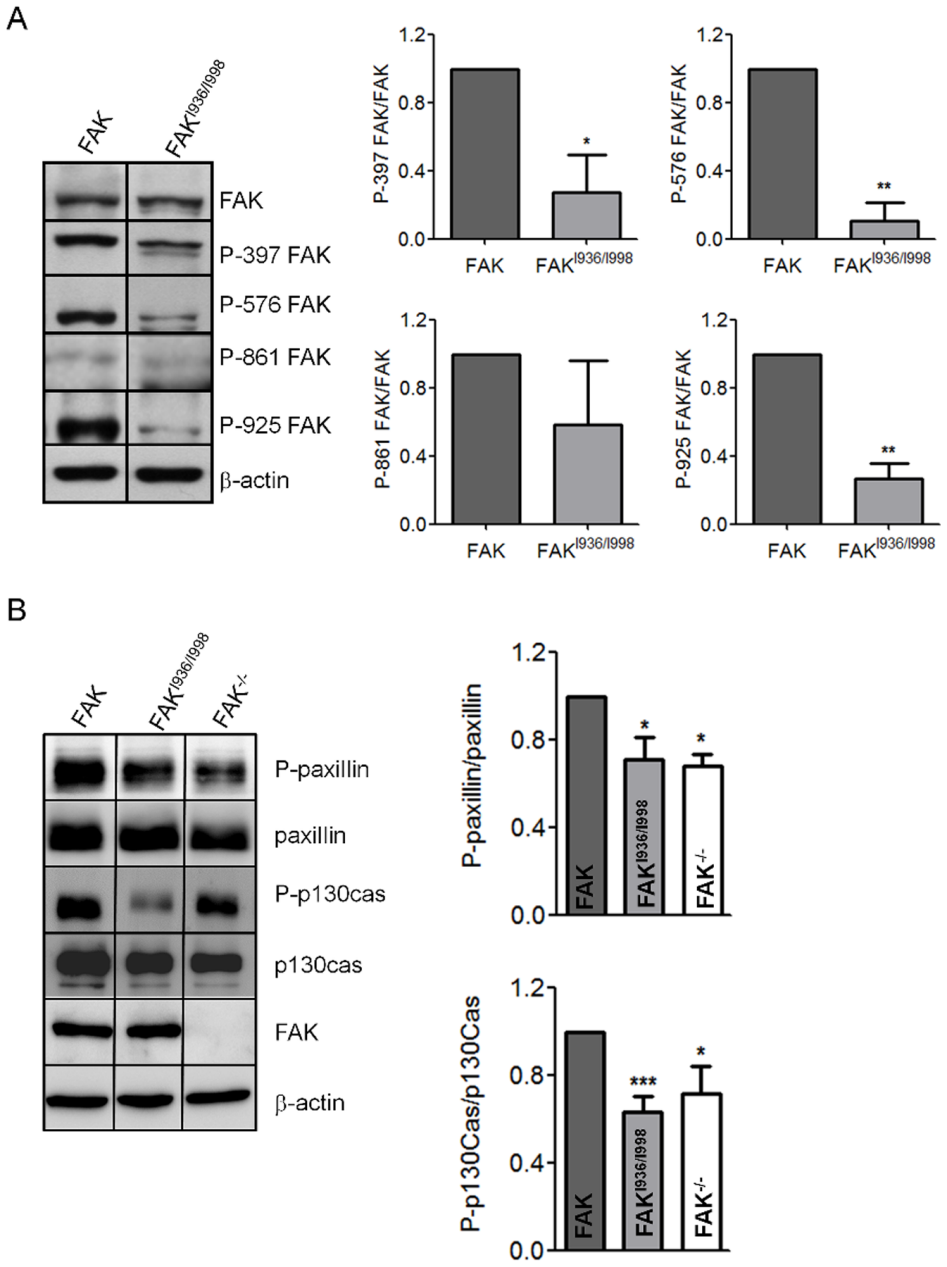
Phosphorylation status of FAK and FAK substrates in FAK^-/-^ cells re-expressing wild-type FAK and FAK^I936/I998^. (A) Graphs show decreased FAK phosphorylation at Tyr^397^, Tyr^576^ and Tyr^925^ in FAK^I936/I998^ cells compared to wild-type FAK cells (*p<0.013, **p<0.0002; n = 4 to 6 independent experiments). (B) Representative Western blots showing the phosphorylation states of paxillin and p130Cas in FAK^-/-^, FAK and FAK^I936/I998^-expressing cells. Graphs show decreased phosphorylation of paxillin in both FAK^I936/I998^ cells and FAK^-/-^ cells (*p<0.03) compared to wild-type FAK cells (P = 0.0097, F = 6.165) and decrease phosphorylation of p130CAS in FAK^I936/I998^ cells (***p<0.001) and FAK^-/-^ cells (*p<0.05) compared to wild-type FAK cells (p = 0.0004, F = 12.68; n = 3 to 8 independent experiments).

To analyse the effects of the FAK^I936/I998^ mutation on the state of phosphorylation at tyrosine sites, Western blots were done using phosphospecific Abs that recognize phosphorylation at Tyr^397^, Tyr^576^, Tyr^861^ and Tyr^925^ of FAK. Phosphorylation at Tyr^861^ was only slightly affected by mutations of FAK in the FAT domain whereas phosphorylation at Tyr^397^, Tyr^576^ and Tyr^925^ was significantly decreased by 72%, 89% and 72% respectively (Figure 2A). FAK kinase activity also regulates downstream substrates including paxillin and p130Cas. In order to characterize the effect of inappropriate FAK location on activation of paxillin and p130Cas, Western blot analysis using Abs against paxillin and p130Cas was done. The results show that FAK mutated at I936/I998 significantly reduced paxillin phosphorylation by 29% compared to wild-type FAK which is comparable to the 32% reduction observed in FAK^-/-^ cells (Figure 2B). On the other hand, p130Cas phosphorylation was reduced by 38% in FAK^I936/I998^ cells as compared to wild-type FAK cells, which is slightly higher than the 28% decrease observed in FAK^-/-^ cells (Figure 2B). This indicates first that FAK-paxillin interaction is crucial for maximal phosphorylation of these substrates of FAK. Moreover, the decrease in p130Cas phosphorylation may be accounted for the lack of FAK localization at FAs, based on our previous observations showing that p130Cas is mainly phosphorylated in FAs of fibroblasts.

### Altered FAK-paxillin interactions affect focal adhesion dynamics

**Figure 3:**
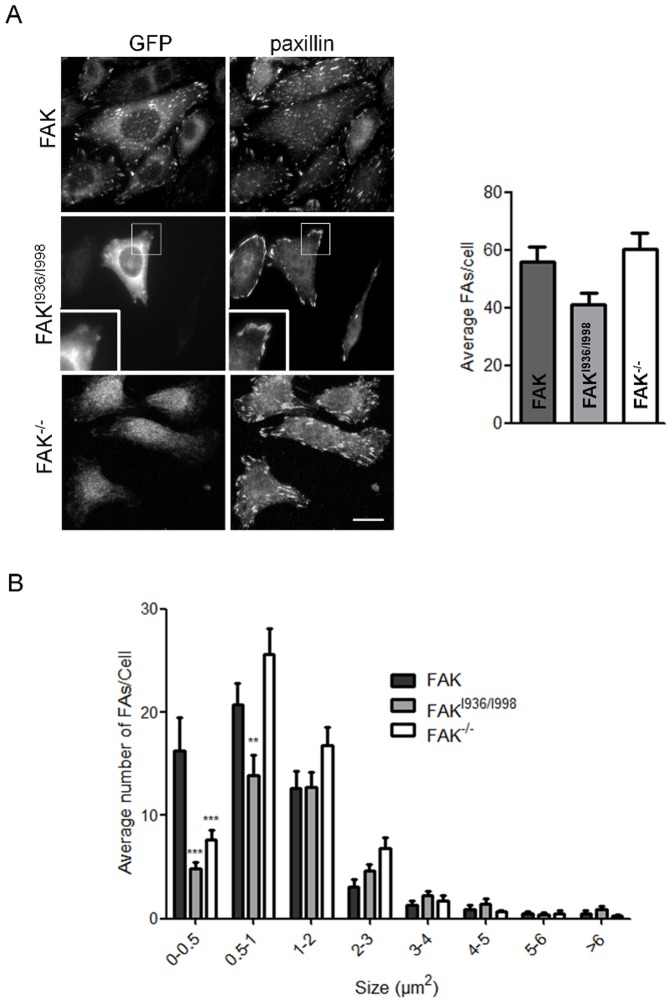
Quantification of focal adhesions in FAK, FAK^I936/I998^ and FAK^-/-^ cells. (A) Confocal images from fixed FAK^-/-^ cells expressing or not wild-type or mutant FAK-GFP and immunostained for paxillin. Evaluation of paxillin-containing FAs reveals no significant decrease in the number of FAs in FAK^I936/I998^ cells as compared to FAK^-/-^ and FAK cells (P = 0.0602, F = 2.958; n = 3 independent experiments with 15 to 28 cells and more than 600 FAs counted per condition). Scale bar, 20 μm. (B) Size-distribution of FAs shows a clear deficit of sub-micron sized FAs in FAK^I936/I998^ (0<FA<0.5 μm^2^ ***p<0.001; 0.5<FA<1 μm^2^ **p<0.01) and FAK^-/-^ cells (0<FA<0.5 μm^2^ ***p<0.001) compared to FAK cells.

**Figure 4:**
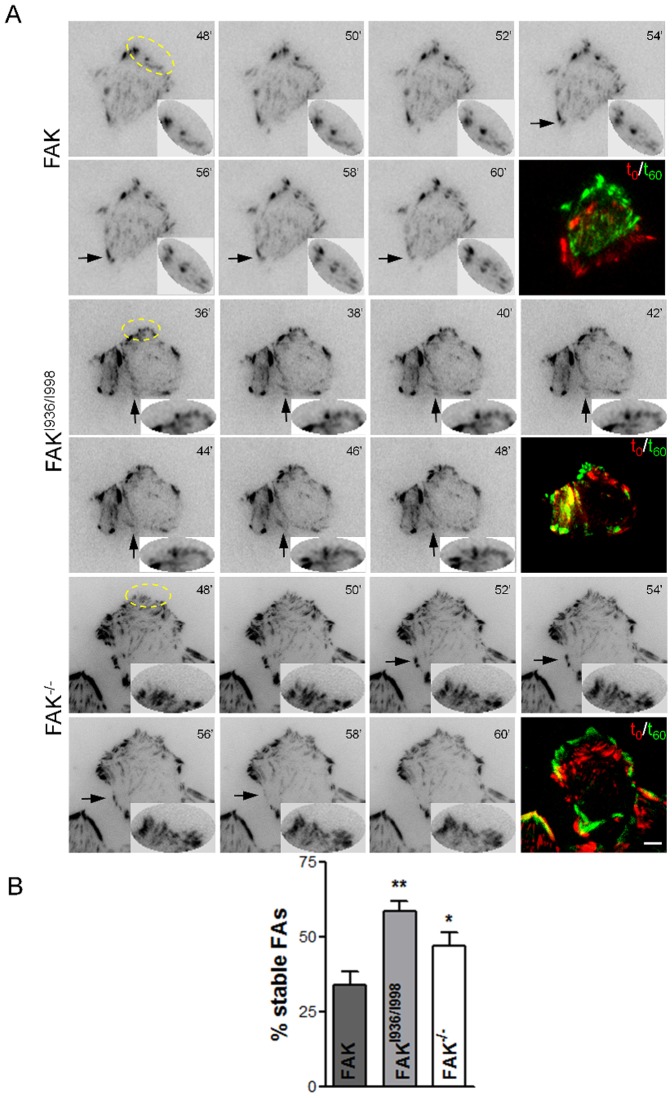
Effects of FAK^I936/I998^ expression on FA dynamics. (A) Representative TIRF images of FAK, FAK^I936/I998^ and FAK^-/-^ cells. Formation of nascent adhesions at the front and dissociation of FAs at the rear (arrows) in FAK, FAK^I936/I998^ and FAK^-/-^ cells transfected with CFP-paxillin. Arrows point to disassembling FAs at the trailing edge. Insets are magnified views of nascent adhesions over time in the yellow ellipses. Images were acquired at 1 min intervals for 1 h (scale bar, 5 μm) and representative images from the time-lapse sequence are shown at 2 min intervals. Colour images represent the superposition of images taken at t_0_ (red) and t_60_ (green). (B) Percentages of stable FAs were calculated from images in (A) as the number of FAs in FAK, FAK^I936/I998^ and FAK^-/-^ cells at t = 60 min compared to t = 0. (P = 0.0055, F = 9.145; *p<0.03, n = 3 independent experiments).

We and others have demonstrated that FAK phosphorylation is essential for the regulation of FA turn-over [25], [26]. Numerous studies have also confirmed the implication of paxillin phosphorylation in this process. As FAK mutated at I936 and I998 decreases both FAK and paxillin phosphorylation, we investigated whether this could lead to impaired FA dynamics. For this purpose, we evaluated the number and size of FAs in FAK^-/-^ MEFs and MEFs expressing wild-type FAK or FAK^I936/I998^. Cells were seeded on fibronectin-coated coverslips and processed for paxillin immunolabeling (Figure 3A), as paxillin has been shown to be a reliable marker of FAs [27]. We first observed that cells expressing FAK^I936/I998^ display a small, non significant reduction of the number of FAs (41±4) as compared to FAK cells (56±5) and FAK^-/-^ cells (60±6) (Figure 3B). Further analysis of the size distribution of FAs revealed a drastic decrease in submicron-sized FAs, which suggests an impaired mechanism for nascent FA formation and/or an alteration in the FA disassembly process. A similar phenotype, present to a lesser extent, was also observed in FAK^-/-^ cells, with the distribution of FAs being shifted to sizes >1 μm^2^ (Figure 3B). To gain insight into these processes, FAK^-/-^ MEFs and FAK^-/-^ MEFs expressing wild-type FAK or FAK^I936/I998^ were co-transfected with CFP-paxillin, grown to confluence, wounded by scraping with a pipette tip, and tracked over 1 h using live cell TIRF microscopy. Time lapse acquisition in TIRF mode of images of FAK^-/-^ cells (Movie S1), FAK cells (Movie S2) and FAK^I936/I998^ (Movie S3) cells showed newly formed nascent FAs at protruding leading edges and disassembly FAs at trailing edges (Figure 4A). It is noteworthy that both FA formation at the cell front and FA disassembly at the cell rear were impaired in FAK^I936/I998^ cells compared to FAK cells. Quantification of FA dynamics shows that 35% of FAs remained stable during the observation period in wild-type FAK cells whereas this percentage increased to 47% in FAK^-/-^ cells and to 58% in FAK^I936/I998^ cells (Figure 4B). These data confirm that FAK-paxillin interaction plays a critical role in regulating FA turnover.

### Altered FAK-paxillin interactions disrupt cell adhesion and migration

**Figure 5:**
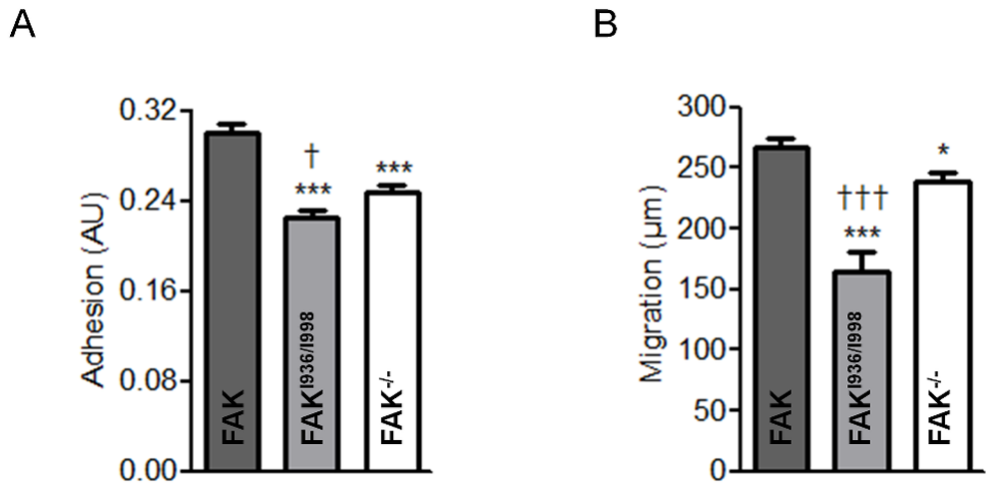
Effect of FAK^I936/I998^ expression on adhesion and migration. (A) For adhesion assay, cells were seeded in fibronectin-coated wells and allowed to adhere for 1 h before quantification. (P<0.0001, F = 28.96; 3 independent experiments done in sextuplicates). ***p<0.0001 versus FAK cells, ^†^p<0.02 versus FAK^-/-^ cells. (B) For migration assays, confluent cell monolayers were wounded and cells were allowed to migrate for 8 h. Images were taken at t = 0 and t = 8 h, and the distance covered by migrated cells was evaluated. (P<0.0001, F = 21.57; 3 independent experiments done in triplicates). ***p<0.0001 versus FAK cells, *p<0.02 versus FAK cells, ^††^p<0.005 versus FAK^-/-^ cells.

We next investigated whether FAK^I936/I998^ impaired cell adhesion and migration. Adhesion assays revealed that MEFs expressing FAK^I936/I998^ were less efficient in adhering on fibronectin-coated dishes compared to MEFs expressing wild-type FAK and FAK^-/-^ cells (Figure 5A). Next, wound healing experiments were done to evaluate how the lack of FAK localization at FAs affects cell migration (Figure 5B). To this end, FAK^-/-^, wild-type FAK and FAK^I936/I998^ cells were grown to confluence, wounded by scraping with a pipette tip, and images of wounded areas were taken at t = 0 and after 8 h of migration. Distances of migration during this time were then determined for each cell line and showed that wild-type FAK cells (268±6 μm) migrated significantly further than FAK^-/-^ cells (243±8 μm) and FAK^I936/I998^ cells (164±17 μm). Thus, expression of FAK^I936/I998^ decreased migration speed by 30% compared to control FAK^-/-^ cells and by 40% compared to wild-type FAK cells (Figure 5B). Taken together, given that FAK^I936/I998^ cells display significantly reduced adhesion and migration compared to FAK^-/-^ cells, this suggests an alteration in the pathways involved in invasion processes that could be linked to the displacement of key partners out of FAs.

### Decreased cell invasion of FAK^I936/I998^ cells

**Figure 6:**
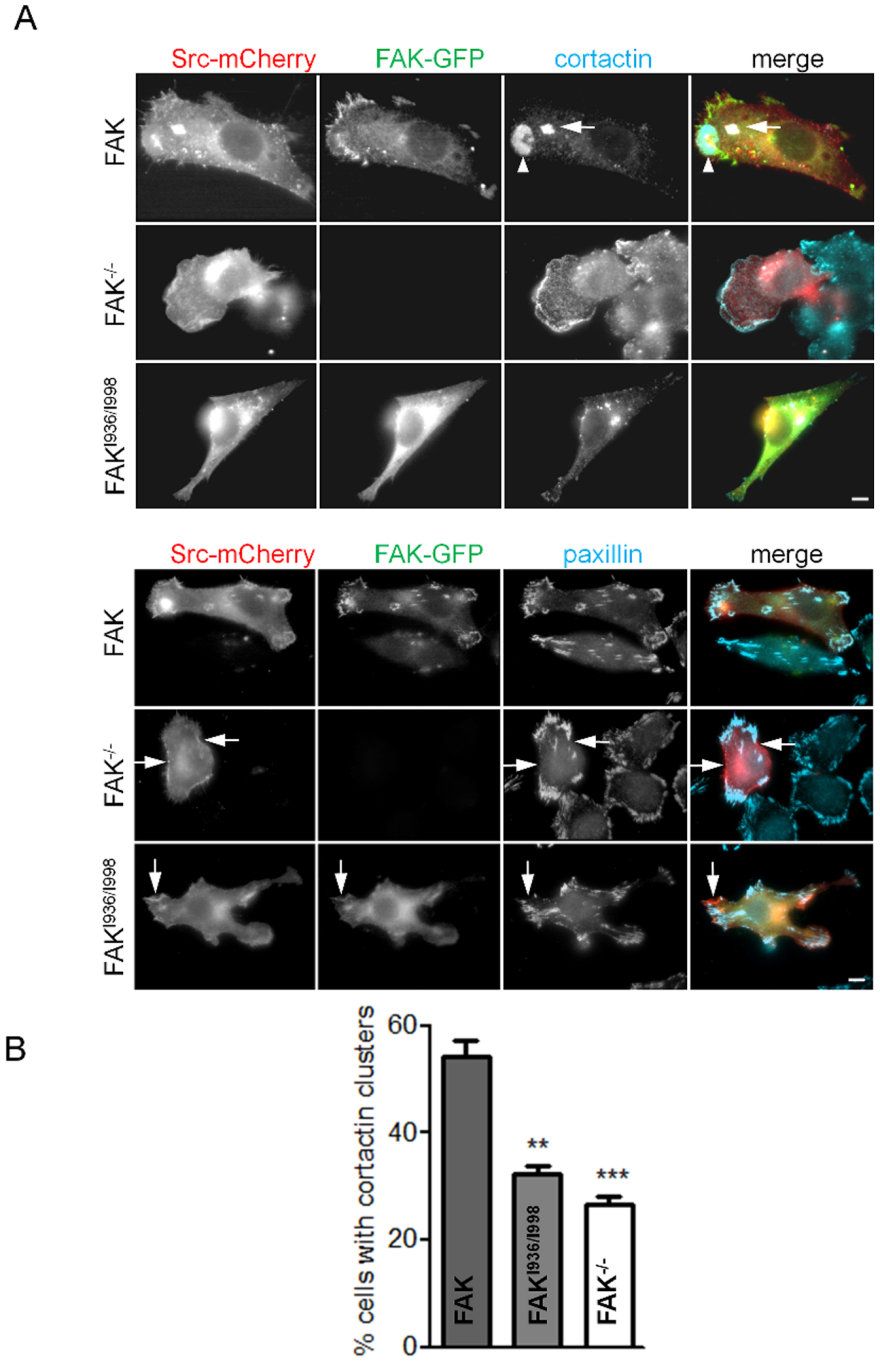
Cortactin and paxillin distributions in FAK^-/-^, wild-type FAK and FAK^I936/I998^ cells co-transfected with active Src. (A) Fluorescence images taken at the base of fixed cells expressing wild-type or mutant GFP-FAK and mCherry-Src^Y530F^ and immunostained for cortactin (upper panel) or paxillin (lower panel). Note the presence of cortactin clusters organized in rosette-like (arrowhead) or dot-like (arrow) structures characteristic of Src transformation in FAK cells and the absence of such structures in FAK^I936/I998^/Src^Y530F^ cells. Note also the membrane localization of Src^Y530F^ in FAK^I936/I998^ and FAK^-/-^ cells (arrows). Scale bars, 5 μm. (B) Quantification of the percentage of cells having cortactin clusters at their base in the different conditions above. Note the reduced amount of cells with cortactin clusters in FAK^-/-^ and FAK^I936/I998^ expressing cells. (P = 0.0003, F = 40.92; **p<0.003, ***p<0.002 versus FAK cells, 3 independent experiments with approximately 100 cells counted for each condition).

**Figure 7:**
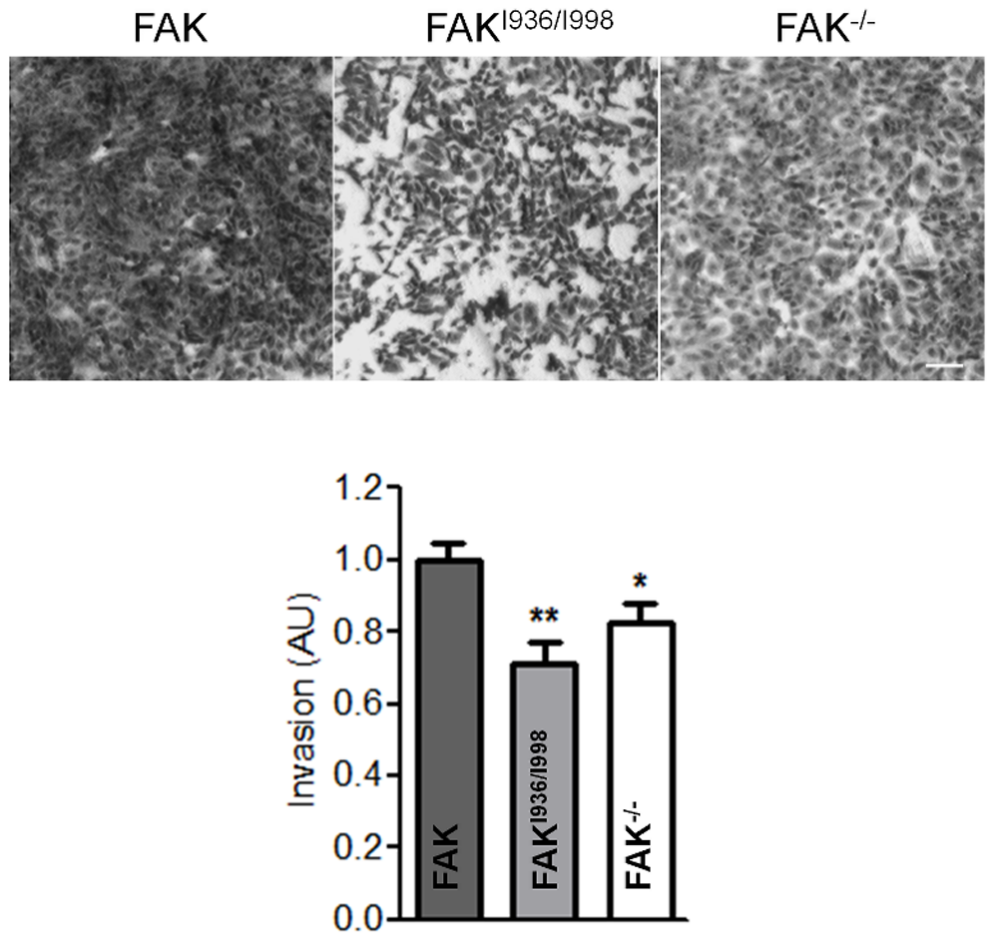
Invasion of FAK^-/-^, wild-type FAK and FAK^I936/I998^ cells co-transfected with active Src. Transfected cells were seeded in Matrigel-coated Boyden chambers and allowed to invade for 24 h. Invading cell nuclei were stained with crystal violet and ODs were measured at 595 nm. Bright field images were taken at 4× magnification. Scale bar, 100 μm. (P = 0.0030, F = 7.795; *p<0.03, **p<0.002 versus wild-type FAK cells from 3 independent experiments done in triplicates).

It is well known that permanently active kinases like vSrc or Src^Y530F^ have transforming capabilities leading to aberrant cell morphology in culture. This is especially well described in Src-transformed mouse fibroblasts where Src induced the formation of specialized structures dedicated to invasion processes. We therefore utilized this property to evaluate the effect of FAK mutation on cell invasion. Thus, FAK^-/-^ MEFs and MEFs expressing wild-type FAK or FAK^I936/I998^ were transfected with mCherry-Src^Y530F^. Src-induced transformation of FAK cells resulted in the appearance of invasive structures at the cortex that were characterized by cortactin enrichment, visible as clusters (Figure 6A top panels, arrows) and/or rosettes (Figure 6A top, arrowheads). Quantification of the number of cells displaying such structures revealed a 40 to 50% decrease in FAK^I936/I998^ and FAK^-/-^ cells respectively as compared to FAK cells (Figure 6B). It has been reported that Src localizes to FAs via binding to FAK at Tyr^397^ [28]. Indeed, as observed by the reduction of Src-paxillin co-localization (Figure 6A bottom panels) in FAK^-/-^ or FAK^I936/I998^ cells, mCherry-Src^Y530F^ targeting to FAs appears inhibited, but remains in other cell membrane (arrows) and cytoplasmic regions. Thus, one of the effects of the FAK^I936/I998^ mutation may be the displacement of Src from active sites. The invasive properties of Src-transformed MEFs were then evaluated in invasion assays in which the cells degrade and migrate through Matrigel. Using this assay, invasion of both FAK^-/-^/Src^Y530F^ and FAK^I936/I998^/Src^Y530F^ cells were significantly less than for wild-type FAK/Src^Y530F^ cells (Figure 7). These results show that the invasive phenotype triggered by active Src^Y530F^ expression is inhibited in FAK^-/-^ or FAK^I936/I998^ cells.

### Gain of function of FAK^I936/I998^

**Figure 8:**
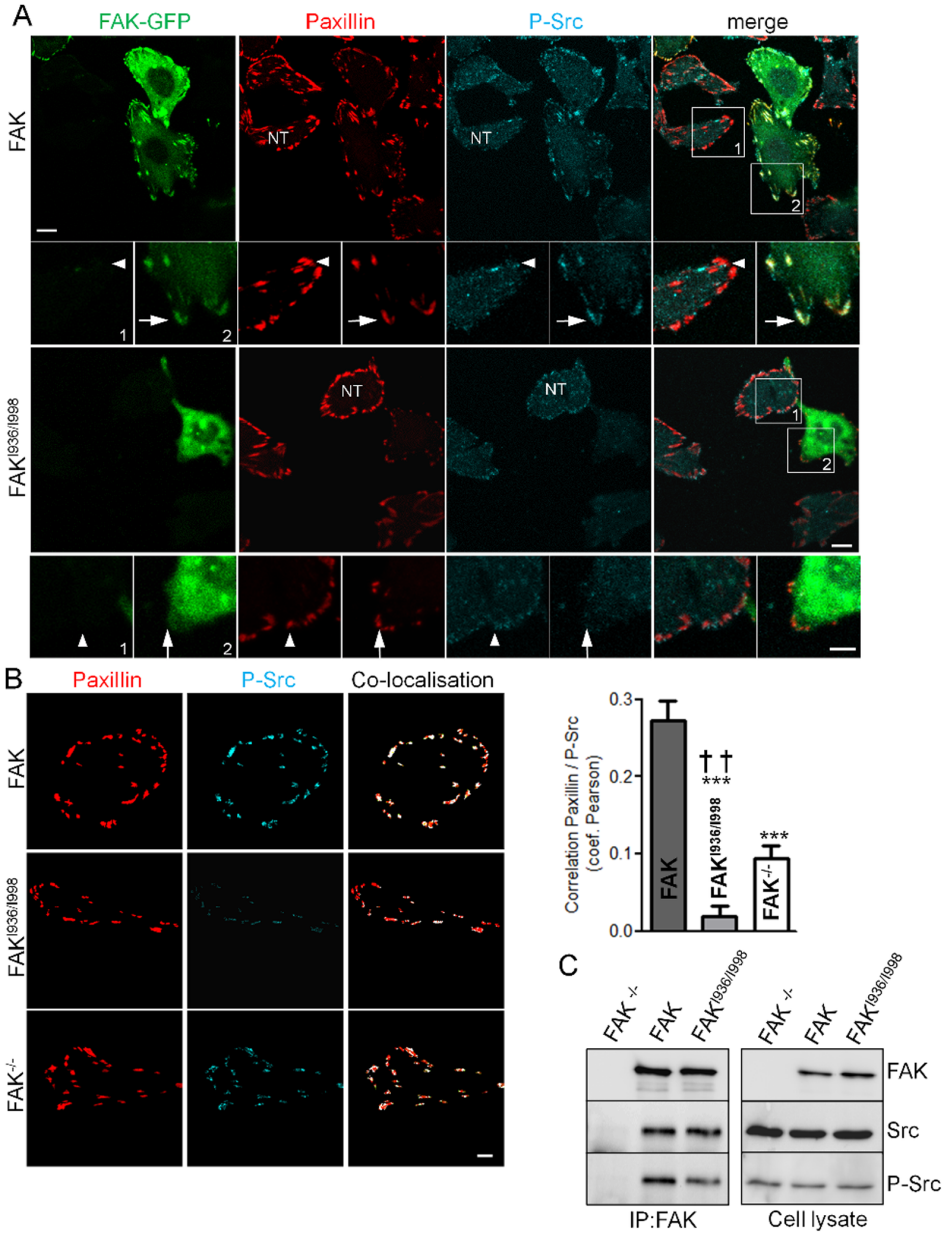
P-Src distribution and interaction in FAK^-/-^, wild-type FAK and FAK^I936/I998^ cells. (A) Confocal images of fixed FAK^-/-^cells expressing or not (NT, non-transfected cells) wild-type or mutant FAK-GFP (left panel) and immunostained for paxillin (middle panel) or Src phopsphorylated at tyr 416 (right panel). Note the high level of P-Src at FAs (arrows), identified by paxillin staining, in GFP-FAK cells (upper panel) and the lack of P-Src staining at FAs in FAK^I936/I998^-GFP cells (lower panel). Note also the low level of P-Src at FAs in non-transfected (NT) FAK^-/-^ cells (arrowheads). Scale bar, 10 μm, insert scale bar, 5 μm. (B) Analysis of the localisation pattern of P-Src and paxillin in the different conditions above. FAs were first segmented and then subjected to co-localisation analysis. Co-localised pixels between paxillin staining (left) and P-Src staining (middle) appear in white on the colocalised image (right) Scale bar, 10 μm.Quantification using Pearson's coefficient (which describes the extent of overlap between image pairs) reveals a significant reduction of correlation in paxillin and P-Tyr416-Src images for both FAK^I936/I998^ and FAK^-/-^ cells compared to FAK cells (P<0.0001, F = 39.25; ***p<0.0001, n = 3 independent experiments with 15 to 27 cells analyzed) and for FAK^I936/I998^ cells compared to FAK^-/-^ cells (^††^p<0.005, n = 3 independent experiments with 21 to 27 cells analyzed). (C) Representative blots showing wild-type FAK and FAK^I936/I998^ immunoprecipitated using anti-FAK Ab and blotted for FAK, Src and phospho-Src. The expression level of proteins in the corresponding cell lysate is shown.

**Figure 9:**
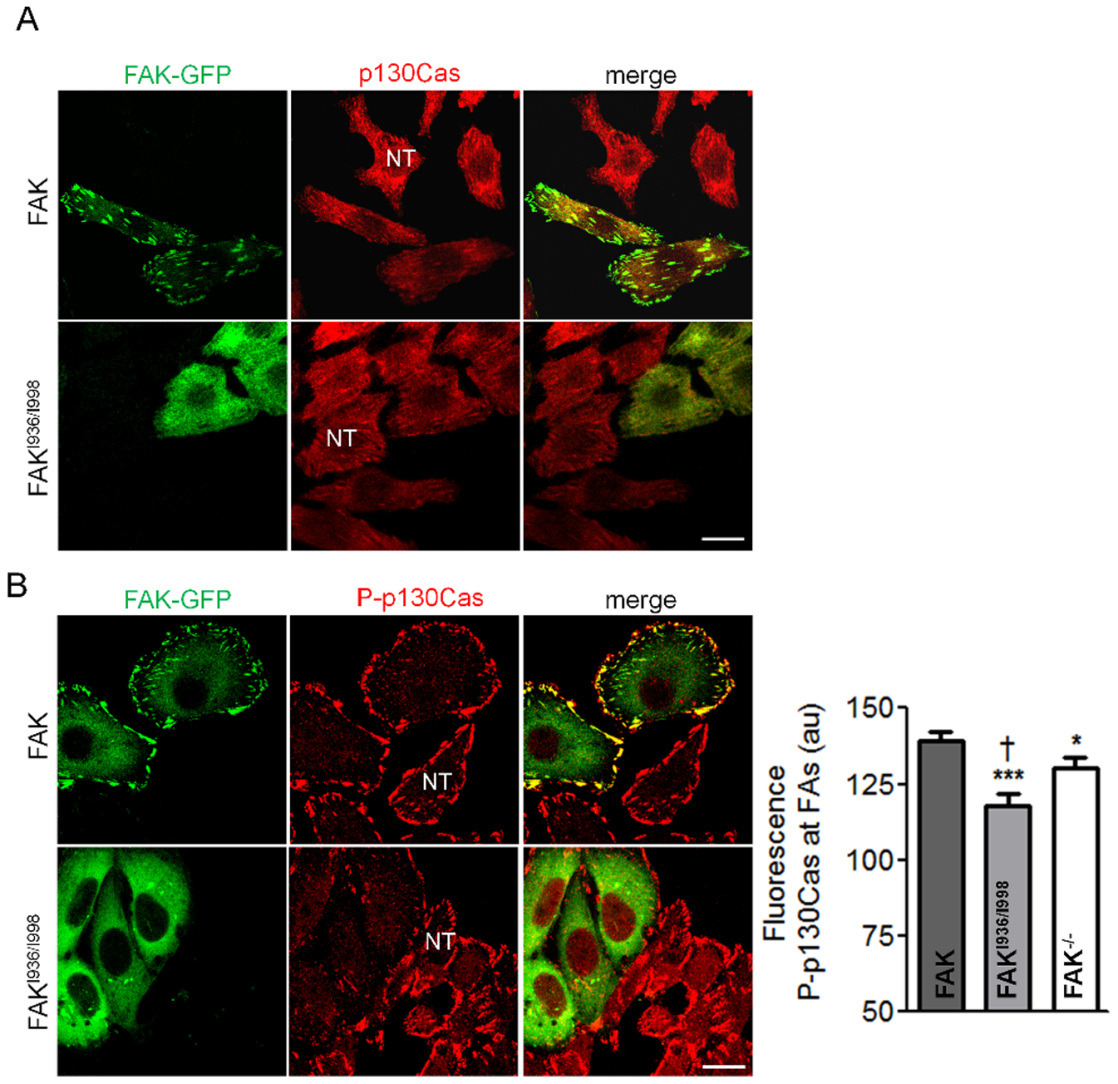
p130Cas distribution in FAK^-/-^, wild-type FAK and FAK^I936/I998^ cells. (A) Confocal images of fixed FAK^-/-^cells expressing or not (NT, non-transfected cells) wild-type or mutant FAK-GFP (left panel) and immunostained for p130Cas (right panel). Note the localization of p130Cas at FAs in both FAK^-/-^, FAK^I936/I998^ and FAK cells. Scale bar, 20 μm. (B) Confocal images of fixed FAK^-/-^cells expressing or not (NT, non-transfected cells) wild-type or mutant FAK-GFP (left panel) and immunostained for P-p130Cas (right panel). Note the high level of P-p130Cas at FAs in GFP-FAK cells (upper panel) and the lack of P-p130Cas staining at FAs in FAK^I936/I998^-GFP cells (lower panel). Scale bar, 20 μm. Quantification of the mean fluorescence intensity at FAs shows a significant reduction of P-p130Cas staining for both FAK^I936/I998^ compared to FAK and FAK^-/-^ cells and for FAK^-/-^ cells compared to FAK cells (P = 0.0005, F = 9.434; ***p<0.0005, *p<0.05 and ^†^p<0.05 respectively, n = 3 independent experiments with 12 to 15 cells analyzed and more than 300 FAs analyzed for each condition).

In our functional tests, we observed a decrease in adhesion, migration and invasion of FAK^I936/I998^-expressing cells often superior to those observed in FAK^-/-^ cells. This led us to suppose that FAK^I936/I998^ may have additional functions by sequestrating key signalling molecules outside FAs. As FAK has been proposed to mediate Src targeting to FAs [28], we began to investigate this property by analysing the activation state of Src at FAs in our cells lines. To do so, immunochemistry experiments were done using antibodies directed against paxillin, to identify FAs and the phosphorylated form of Src at Tyr-416, a hallmark of Src activation states [29], [30]. The intensity of paxillin fluorescence at FAs was independent of FAK^I936/I998^ expression level (Figure S2). We found that Src phosphorylation at FAs is reduced, but not supressed, in non-transfected cells and in cells expressing FAK^I936/I998^ as compared the FAK-expressing cells (Figure 8A). To quantify the degree of Src activation at FAs, FAs were first segmented and then subjected to colocalisation analysis using Pearson's coefficient, which calculates the degree of overlap between paired images (Figure 8B). It appears that Src activation was lower in FAK^I936/I998^ and FAK^-/-^ cells compared to FAK-expressing cells. Moreover, the increased severity of Src inactivation in cells expressing FAK^I936/I998^ compared to FAK^-/-^ cells (Figure 8B), suggests that FAK^I936/I998^ may sequester Src outside FAs. To verify our hypothesis, co-immunoprecipitation experiments were performed. The results show that FAK^I936/I998^ was able to precipitate Src in a manner comparable to FAK WT although Src activity state was reduced in the complex (Figure 8C). Therefore, this suggests that FAK-Src complex pre-exists in the cytosol thus further validating the sequestration property of the FAK mutant. Our Western-blot experiments revealed also that p130Cas was slightly reduced in FAK^I936/I998^ cells compared to FAK^-/-^ cells (Figure 2). Because FAK has been shown to mediate p130Cas phosphorylation either directly or via formation of a complex with Src at FAs, we assayed whether lack of FAK localisation at FAs alter p130Cas phosphorylation at these sites. Immunohistochemistry experiments revealed that p130Cas expression at FAs was similar in both FAK^-/-^, FAK and FAK^I936/I998^ cells (Figure 9A) while p130Cas phosphorylation was reduced in FAK^I936/I998^ cells as compared to FAK cells but also to FAK^-/-^ cells (Figure 9B). Taken together, these data demonstrate that FAK^I936/I998^ displays gain of function effects, thus leading to an enhanced reduction of adhesion and migration properties.

## Discussion

FAK is involved in many aspects of the cancer process and clinical studies have revealed a clear link between FAK expression and/or phosphorylation and cancer aggressiveness. Therefore, the current development of strategies aimed at inhibiting FAK is of potential therapeutic interest. While classical inhibitors of the kinase domain of FAK are effective in animal models, a promising alternative may be to inhibit the scaffolding function of FAK, thereby preventing interaction of FAK with its various binding partners. As many functions of FAK are related to the efficient localization of FAK at FAs, we used site directed mutagenesis to disrupt the two paxillin binding sites in the FAT domain. We found that mutations at Ile^936^, localised in the hydrophobic patch 2 (HP2) and Ile^998^, localised in the hydrophobic patch 1 (HP1) of the FAT domain, completely abolished both FAK-paxillin interaction and targeting of FAK to FAs in fibroblasts. These mutations have been previously used *in-vitro* to test the interaction between the N-terminal domain of paxillin (1–313) and the C-terminal domain of FAK (904-1052) [13]. Here, we characterized the effect of these mutations in living cells having full length paxillin and FAK and report that FA dynamics were altered, cell adhesion disrupted and migration and invasion processes decreased.

Previous studies have already shown that specific mutations of the FAT domain altered FAK-paxillin interactions. Inhibition of paxillin binding to HP1 could be done with the triple mutation E949A/K956A/R963A into the α-helices 2/3 [31]. In this study, when both paxillin binding sites are deleted by the additional mutation of I937A on HP2, FAK localized to adhesions in only 10% of cells, contrarily to the complete delocalization of FAK observed in our study. The authors suggested that, because both paxillin binding sites were mutated, the residual fraction of FAK at FAs was due to FAK targeting by talin in agreement with early deletion studies identifying a 48 amino-acid sequence in the Ct region of FAK (952–1012) necessary for binding to talin [32]. As this sequence includes I998, mutation at this site may potentially alter FAK-talin interaction. Indeed, by combining the triple mutation E949A/K956A/R963A with both I937A/I998A mutations, these authors observed a complete loss of FAK at FAs. Nevertheless, as seen in Figure S1A, talin is correctly localized at FAs in FAK cells but also in FAK^I936E/I998E^ and FAK^-/-^ cells. Moreover, as talin interacts with both FAK and FAK^I936/I998^ (Figure S1B), I998 mutation did not alter the ability of FAK to form a complex with talin. Taken together, these results show that talin may not be involved in FAK targeting to FAs as previously thought. This is in agreement with a recent study mapping FAK-talin interaction to a FAK 1011–1042 domain and demonstrating that during nascent FAs formation, its indeed FAK that targets talin to these sites and not the contrary [24].

Formerly, it has been shown that chicken embryo cells expressing FAK with deletions into the FAT domain like dl853–963 or dl965–1012 were defective for paxillin phosphorylation and adhesion regulation of FAK [15], [33]. Conversely, in these cells over-expression of FAT delayed cell spreading and reduced FAK tyrosine phosphorylation [34] while in astrocytoma, FAT over-expression reduced FAK phosphorylation and invasion in a Boyden chamber Matrigel assay [35] reminding the phenotype observed in our study. The downstream effect of FAK inhibition could be due either to reduced phosphorylation of FAK substrates like paxillin and p130Cas and/or to reduced binding of FAK partners. At least 6 phosphorylatable tyrosines that might potentially bind SH2-containing proteins are present in FAK. Inhibition of FAK targeting to FAs led to reduced phosphorylation of FAK at Tyr^397^, Tyr^576^ and Tyr^925^. In living cells it has been shown, using forced dimerization, that FAK autophosphorylation is essentially intermolecular and requires the presence of the C-terminal targeting region of FAK, presumably because of the local enrichment of FAK at FAs [36]. Therefore, consistently, we observed that lack of FAK localization to adhesion sites dramatically reduces Tyr^397^ phosphorylation. Src binding to FAK promotes further phosphorylation of FAK at additional tyrosines and full catalytic activation of the kinase [37]–[39]. Tyr^576^, which lies within the kinase domain of FAK, is a marker of FAK kinase activity [37]. Thus, the reduced degree of phosphorylation of this site observed in our study could reflect the reduced binding of Src to FAK. This is also consistent with the reduced phosphorylation observed at Tyr^925^, since phosphorylation on this residue is significantly reduced in cells expressing a kinase-defective mutant of Src [40].

Mutation of FAK at Ile^936^ and Ile^998^ led to an overall reduction in the number of small adhesions, especially those with size under 1 μm^2^, which could be due to the reduced phosphorylation of FAK at Tyr^925^ as phosphorylation at this site has been shown to be important for the formation of nascent adhesions [41]. This phenotype was also observed in FAK^-/-^ cells or when cells were transfected with FRNK, the dominant negative form of FAK [42]. Other studies have implicated the phosphorylation state of paxillin in the control of adhesion turn-over. Indeed, using paxillin^-/-^ and paxillin^-/-^ cells expressing either paxillin^Y31F^, paxillin^Y118F^ or paxillin^Y31F/Y118F^, it was demonstrated that paxillin phosphorylation is essential for adhesion turn-over [26]. In addition, *in vitro* studies have shown that FAK phosphorylates paxillin at Tyr 31 and 118 [33], [43], [44]. As FAK^I936/I998^ is unable to bind paxillin, its phosphorylation state is consequently reduced and therefore, our study characterizes a new way to alter FA dynamics via reduction of both FAK and paxillin phosphorylation states. Moreover, binding of p130Cas to FAK is linked to enhanced tyrosine phosphorylation of p130Cas, resulting in the activation of Rac, lamellipodia formation and promotion of cell motility as we and others have previously shown [45]–[47]. Thus, our results suggest that displacing FAK from FAs, which led to reduced paxillin and p130Cas phosphorylation, alters migration via both reduction of FA turnover and lamellipodia formation. It is interesting to note that Ile^936^ and Ile^998^ mutations led to greater decreases in adhesion and migration compared to FAK^-/-^ cells, indicating that FAK^I936/I998^ mutation triggers effects that go beyond FAK removal. Indeed, the global state of p130Cas phosphorylation, as assessed by western-blotting, was slightly reduced in FAK^I936/I998^ cells compared to FAK^-/-^ cells, although this reduction is clearly evident at FAs (Figure 9B). Nevertheless, this reduction did not correlate with a reduction of p130Cas expression level at FAs suggesting that FAK is not a key determinant for p130Cas targeting to FAs. This is in accordance with previous studies showing that p130Cas localizes to FAs via both FAK dependent and independent manner [48]. Indeed, in FAK^-/-^ cells, p130Cas localizes to FA via a mechanism that may implicate the “Cas-family C-terminal homology” (CCH) domain which can adopt a tertiary structure similar to the FAT domain of FAK, and/or bind to the LIM protein Ajuba for targeting to nascent adhesion [49]. The gain of function effect of FAK^I936/I998^ was confirmed by analysing the cellular location of Src, another key binding partner of FAK. We show that Src activation state at FAs is decreased in FAK^I936/I998^-expressing cells as compared to FAK^-/-^ cells. This suggests that the mutated form of FAK is able to sequester key binding partners outside FAs, necessary for FA disassembly. In support of this finding we show that FAK^I936/I998^ bound to Src and therefore, because FAK^I936/I998^ is not localized at FA, this result suggests that FAK^I936/I998^ form a complex with Src within the cytosol.

When fibroblasts were transfected with the constitutively active form of Src, we observed a transformed phenotype characterized by the presence of invadopodia, as previously described [28]. We found that inhibiting FAK localization to FAs reduces the number of cells displaying invadopodia close to the value observed for FAK^-/-^ cells (Figure 6B). This leads to a less invasive phenotype as observed by the reduced invasion through Matrigel (Figure 7), in agreement with prior studies demonstrating that FAK is required for cell invasion through Matrigel in v-Src-transformed FAK^-/-^ fibroblasts [47]. In this latter study, cell invasion was linked to the formation of a complex containing FAK, Src and p130Cas that is necessary for increased MMP activity. More recently, in several cancer cell lines, MT1-MMP-induced matrix degradation at FAs has been described for which the association of the FAK–p130Cas complex at these sites is required [50]. Therefore, alteration of FAK targeting to FAs, which affects p130Cas phosphorylation, could be responsible for the reduced invasion observed in FAK^I936/I998^ cells (Figure 7). We also show that Src localization to paxillin-containing structures is enhanced by FAK targeting at FAs (Figure 6A). Using time-lapse video of GFP-tagged vinculin, it has been found that Src-transformed NIH3T3 cells assemble podosomes at FAs, therefore supporting the notion that podosomes might be initiated at these sites [51].

In summary, we have shown that inhibiting FAK-paxillin interactions led to altered FAK localization at FAs which in turn results in reduced phosphorylation of FA proteins and impaired adhesion, migration and invasion processes. Our study also demonstrates that the effects of inhibiting FAK targeting to FAs appears to be greater that complete knockout of FAK. This supports that targeting the FAK “interactor” instead of the FAK kinase domain may well be a valuable strategy for the search of novel FAK inhibitors to treat metastatic cancer.

## Supporting Information

Figure S1 **Talin distribution and interaction in FAK^-/-^, wild-type FAK and FAK^I936/I998^ cells.** (A) Confocal images from fixed cells expressing wild-type or mutant FAK and immunostained for talin (red). Note the presence of talin at FAs in both FAK^-/-^, FAK^I936/I998^ and FAK cells. Scale bar, 20 μm. (B) Representative blots showing wild-type FAK and FAK^I936/I998^ immunoprecipitated using anti-talin Ab and blotted for FAK and talin. The expression level of proteins in the corresponding cell lysate is shown.
(TIF)

Figure S2 **Paxillin distribution in FAK^I936/I998^ cells.** Confocal images from fixed cells expressing FAK^I936/I998^ and immunostained for paxillin (red). Note the equal expression level of paxillin at FAs in both FAK^I936/I998^ and FAK^-/-^, cells. Scale bar, 20 μm.
(TIF)

Movie S1 Fluorescence image sequence of a FAK^-/-^ fibroblast expressing CFP-paxillin. TIRF images are taken at 1 min interval for 1 hour.
(AVI)

Movie S2 Fluorescence image sequence of a FAK^-/-^ fibroblast expressing wild-type FAK and CFP-paxillin. TIRF images are taken at 1 min interval for 1 hour.
(AVI)

Movie S3 Fluorescence image sequence of a FAK^-/-^ fibroblast expressing FAK^I936/I998^ and CFP-paxillin. TIRF images are taken at 1 min interval for 1 hour.
(AVI)
