# Supplementary figures and images for: Altering FAK-Paxillin Interactions Reduces Adhesion, Migration and Invasion Processes
Source: PLoS One. 2014 Mar 18;9(3):e92059. doi: 10.1371/journal.pone.0092059 (PMC3958421; doi:10.1371/journal.pone.0092059)

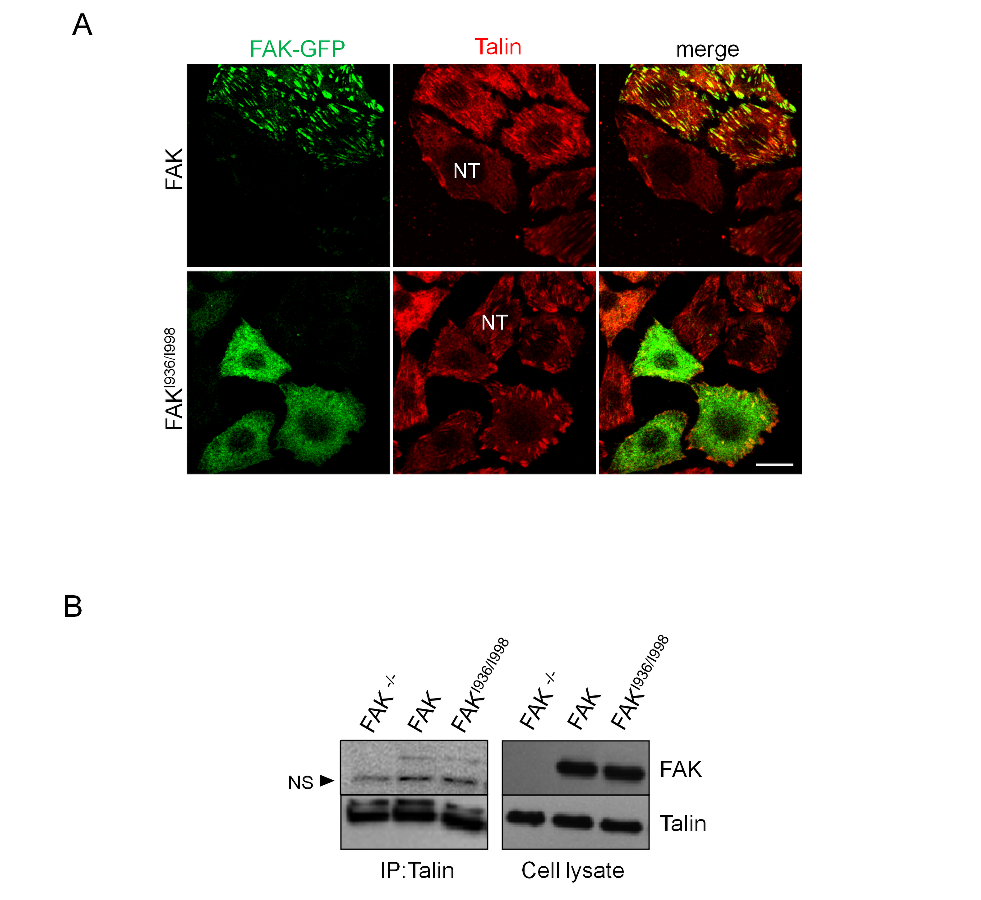

Supplement: Figure S1 — Talin distribution and interaction in FAK-/-, wild-type FAK and FAKI936/I998 cells. (A) Confocal images from fixed cells expressing wild-type or mutant FAK and immunostained for talin (red). Note the presence of talin at FAs in both FAK-/-, FAKI936/I998 and FAK cells. Scale bar, 20 μm. (B) Representative blots showing wild-type FAK and FAKI936/I998 immunoprecipitated using anti-talin Ab and blotted for FAK and talin. The expression level of proteins in the corresponding cell lysate is shown. (TIF) [file pone.0092059.s001.tif]

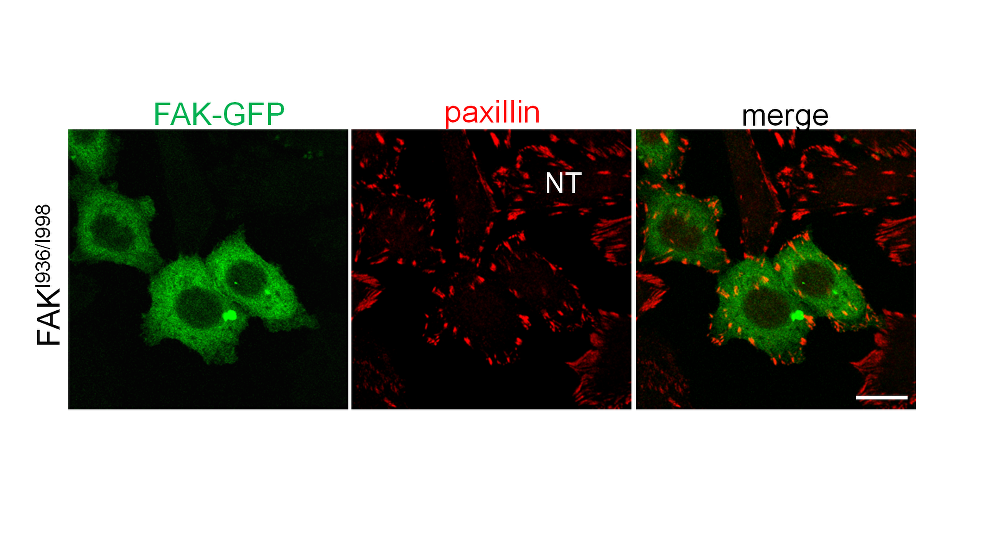

Supplement: Figure S2 — Paxillin distribution in FAKI936/I998 cells. Confocal images from fixed cells expressing FAKI936/I998 and immunostained for paxillin (red). Note the equal expression level of paxillin at FAs in both FAKI936/I998 and FAK-/-, cells. Scale bar, 20 μm. (TIF) [file pone.0092059.s002.tif]
